# Prognostic and clinicopathological role of high Ki-67 expression in patients with renal cell carcinoma: a systematic review and meta-analysis

**DOI:** 10.1038/srep44281

**Published:** 2017-03-13

**Authors:** Yongpeng Xie, Luyao Chen, Xin Ma, Hongzhao Li, Liangyou Gu, Yu Gao, Yang Fan, Yu Zhang, Xu Zhang

**Affiliations:** 1 State Key Laboratory of Kidney Diseases, Department of Urology, Chinese PLA Medical School, Chinese PLA General Hospital, Beijing, People’s Republic of China; 2 Medical School, Nankai University, Tianjin, People’s Republic of China

## Abstract

Previous studies have elevated the prognostic value of Ki-67 in renal cell carcinoma (RCC), but the reports are controversial and inconsistent. We conducted a systematic review and meta-analysis to clarify the significance of Ki-67 in RCC prognosis. We systematically searched PubMed, Web of Science, and Embase to identify relevant studies until April 2016. Based on the inclusion and exclusion criteria, 20 studies, including 5,398 patients, were eligible for further analysis. Results showed that high Ki-67 expression in RCC was associated with poor OS (HR = 1.95, 95% CI: 1.44–2.64), CSS (HR = 1.67, 95% CI: 1.47–1.89), and DFS (HR = 2.56, 95% CI: 1.79–3.67). In addition, high Ki-67 expression was significantly associated with TNM stage (III/IV vs. I/II: RR = 2.03, 95% CI: 1.68–2.44), pathological T stage (T3/T4 vs. T1/T2: RR = 1.67, 95% CI: 1.35–2.06), metastasis (yes vs. no: RR = 2.15, 95% CI: 1.77–2.62), and Fuhrman grade (III/IV vs. I/II: RR = 1.77, 95% CI: 1.20–2.60). Our study suggested that Ki-67 was a prognostic marker in RCC. High Ki-67 expression was correlated with poor prognosis and advanced clinicopathological features, and it could serve as a biomarker for disease management.

Renal cell carcinoma (RCC) is one of the most prevalent urological malignancies worldwide^1^,^2^. The incidence of RCC, a highly aggressive disease, has steadily increased over the years. Approximately 30% of patients have metastases at first diagnosis, and another 20% of RCC patients with clinically localized disease will develop metastasis even after curative nephrectomy^3^. Although novel target therapies have been developed, most metastatic RCCs still eventually cause death^4^. Prediction models identifying patients with poor prognosis, who may benefit from early systematic therapy, are greatly needed. To date, the tumor, node, and metastasis (TNM) staging system is a widely used RCC prognostic predictor. However, it is inferior in accurately predicting the prognosis of RCC patients with diverse and complicated tumor backgrounds^5^. Therefore, novel biomarkers that can stratify patients with poor prognosis should be identified to precisely guide clinical decisions.

Ki-67, a proliferation marker that is expressed during the cell cycles of G1, S, G2, and M stages, except G0, is usually detected by immunohistochemical (IHC) staining^6^. Its strict association with cell proliferation and its co-expression with other well-known markers of proliferation indicate a pivotal role in cell division. Several studies recently reported that Ki-67 expression is associated with poor prognosis in several types of cancer^7^,^8^,^9^,^10^. However, the role of Ki-67 in the prognosis of RCC remains inconsistent. Ki-67 was considered an unfavorable prognostic marker in RCC in many studies^11^,^12^,^13^,^14^, but some studies reported that Ki-67 expression is prognostically irrelevant in RCC patients^15^,^16^. To obtain a more precise evaluation of the prognostic and clinicopathological value of Ki-67 expression in RCC, we performed a systematic review and meta-analysis to evaluate the prognostic value of Ki-67 quantitatively and explore the associations of Ki-67 with the clinicopathological features of RCC.

## Results

### Search Results

**Figure 1:**
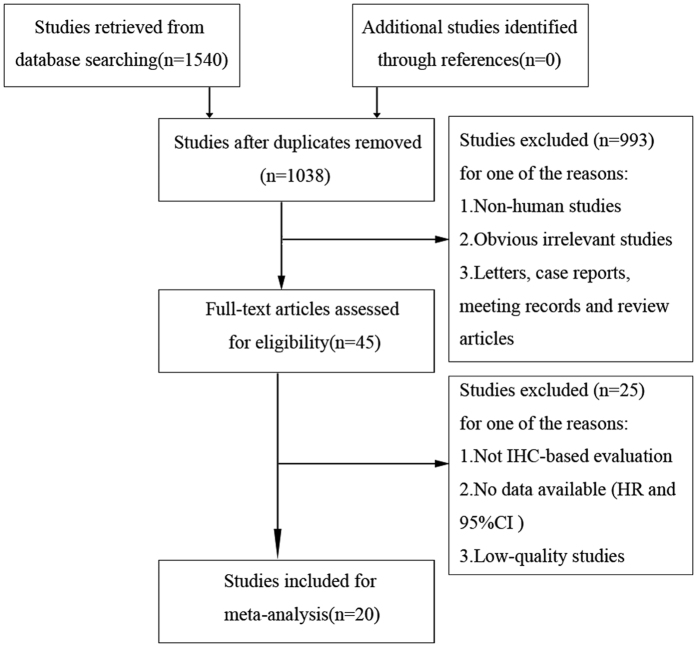
Flow chart of study selection.

Our search strategy initially identified 1540 articles from the primary literature search. A total of 502 duplicate reports were excluded. After screening the titles and abstracts, 993 articles were excluded for various reasons such as non-human studies, letters, case reports, meeting records, reviews, and other obvious irrelevant studies. The remaining 45 articles were evaluated in full text. To avoid the heterogeneity caused by the detection method, studies without IHC evaluation were excluded. The remaining articles were further excluded for several reasons, such as no data available (hazard ratio [HR] and 95% confidence interval [CI]), low-quality studies^17^, and duplicate publication. Finally, 20 articles published from 2000 to 2016 with 5,398 patients satisfied the criteria for meta-analysis^11^,^12^,^13^,^14^,^15^,^16^,^18^,^19^,^20^,^21^,^22^,^23^,^24^,^25^,^26^,^27^,^28^,^29^,^30^,^31^. A flowchart of the study selection process is shown in Fig. 1.

### Characteristics of Studies

The main characteristics of the 20 studies are summarized in Table 1. Patients in these studies were all diagnosed with RCC with different tumor types and received radical or partial nephrectomy. Seven studies originated from the United States, three were from Finland, two were from Germany, two were from China, one was from France, one was from Japan, one was from Norway, one was from Italy, one was from Portugal, and one was from the United Kingdom. Among the studies, five studies were performed to analyze overall survival (OS), 11 studies were conducted to investigate cancer-specific survival (CSS), and six studies reported disease-free survival (DFS). Various clinicopathological data were reported in eight studies (TNM stage in four studies, pathological T stage in eight studies, metastasis in five studies, and Fuhrman grade in eight studies). All studies applied IHC staining to investigate Ki-67 expression. Positive Ki-67 expression was defined using different cutoff values among various studies, so we classified all the cases according to their original studies (positive or negative staining).

### Meta-Analysis

**Figure 2:**
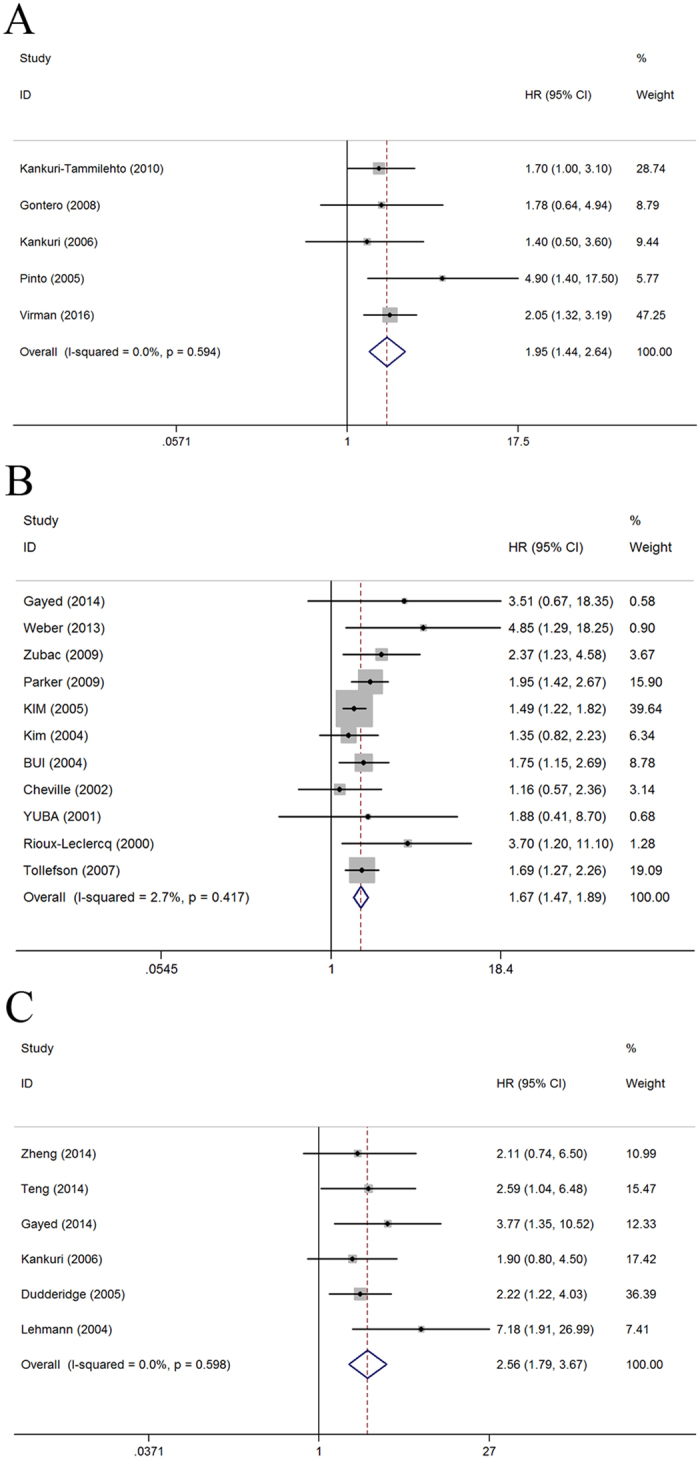
Forest plots of studies evaluating the association between Ki-67 expression and prognostic outcomes of RCC patients: (**A**) effect of Ki-67 overexpression on OS, (**B**) CSS, and (**C**) DFS. HR: hazard ratio; CI: confidence interval; OS: overall survival; CSS: cancer-specific survival; DFS: disease-free survival; RCC: renal cell carcinoma. HR > 1 implies unfavorable prognosis for patients with high Ki-67 expression.

Our meta-analysis demonstrated that high Ki-67 expression in RCC was associated with poor OS (fixed-effect model, HR = 1.95; 95% CI: 1.44–2.64; p < 0.001; *I*^*2*^ = 0.0%, p = 0.594; Fig. 2A), CSS (fixed-effect model, HR = 1.67; 95% CI: 1.47–1.89; p < 0.001; *I*^*2*^ = 2.7%, p = 0.417; Fig. 2B), and DFS (fixed-effect model, HR = 2.56; 95% CI: 1.79–3.67; p < 0.001; I^2^ = 0.0%, p = 0.598; Fig. 2C). To explore the source of heterogeneity, meta-regression analysis on CSS was conducted by ethnicity, tumor extent at time of diagnosis, counting method, cutoff of staining, follow up time. The results showed that ethnicity (p = 0.889), tumor extent at time of diagnosis (p = 0.415), counting method (p = 0.858), cutoff of staining (p = 0.305), and follow up time (p = 0.467) were not significant contributors to heterogeneity. Furthermore, subgroup analysis stratified by ethnicity, tumor extent at time of diagnosis, histopathological subtype, counting method, cutoff of staining, and follow up time was also performed. With regard to ethnicity, high Ki-67 expression was correlated with poor OS (HR = 1.95; 95% CI: 1.44–2.64; p < 0.001), CSS (HR 1.67; 95% CI: 1.47–1.89; p < 0.001), and DFS (HR = 2.63; 95% CI: 1.73–4.01; p < 0.001) in Caucasian patients, as well as with poor DFS (HR = 2.38; 95% CI: 1.18–4.80; p = 0.015) but not with CSS (HR = 1.88; 95% CI: 0.41–8.67; p = 0.417) in Asian patients (Table 2). Regarding the tumor extent, high Ki-67 expression was associated with poor OS (HR 2.01; 95% CI: 1.46–2.77; p < 0.001) and CSS (HR 1.80; 95% CI: 1.52–2.31; p < 0.001) but not with DFS (HR = 2.11; 95% CI: 0.74–6.50; p = 0.178) for all stages of RCC; with poor DFS (HR = 2.63; 95% CI: 1.79–3.85; p < 0.001) but not with OS (HR 1.40; 95% CI: 0.52–3.76; p = 0.504) and CSS (HR = 2.29; 95% CI: 0.85–6.19; p = 0.104) for localized RCC; and with poor CSS (HR = 1.49; 95% CI: 1.22–1.82; p < 0.001) for metastatic RCC. For histopathological subtype, high Ki-67 expression was correlated with poor CSS (HR = 1.67; 95% CI: 1.47–1.89; p < 0.001) and DFS (HR = 3.66; 95% CI: 2.00–6.72; p < 0.001) for ccRCC. With respect to counting method, high Ki-67 expression was correlated with poor OS (HR = 1.86; 95% CI: 1.22–2.82; p = 0.004), CSS (HR 1.66; 95% CI: 1.44–1.90; p < 0.001), and DFS (HR = 2.57; 95% CI: 1.73–3.79; p < 0.001) for eyeball counting, as well as with poor OS (HR = 2.05; 95% CI: 1.32–3.19; p = 0.001) and CSS (HR = 1.73; 95% CI: 1.26–2.38; p = 0.001) for formal counting. In the cutoff of staining subgroup analysis, high Ki-67 expression was correlated with poor DFS (HR = 7.18; 95% CI: 1.91–26.99; p = 0.004) but not with CSS (HR = 1.26; 95% CI: 0.66–2.41; p = 0.476) when the cutoff value was less than 10%. Studies with a cutoff value greater than or equal to 10% showed that high Ki-67 expression was associated with poor OS (HR = 1.86; 95% CI: 1.22–2.82; p = 0.004), CSS (HR = 1.90; 95% CI: 1.45–2.49; p < 0.001), and DFS (HR = 2.36; 95% CI: 1.62–3.43; p < 0.001). Additionally, high Ki-67 expression was associated with poor CSS (HR 1.59; 95% CI: 1.38–1.84; p < 0.001) and DFS (HR = 2.45; 95% CI: 1.54–3.91; p < 0.001) in patients with follow up time less than 60 months; with poor OS (HR = 1.95; 95% CI: 1.44–2.64; p < 0.001), CSS (HR 1.93; 95% CI: 1.22–3.03; p = 0.005), and DFS (HR = 2.73; 95% CI: 1.55–4.82; p = 0.001) in patients with follow up time greater than or equal to 60 months.

In the comprehensive analyses of the role of Ki-67 expression in RCC as a biomarker, we investigated the relationship between elevated Ki-67 expression and clinicopathological characteristics. As shown in Table 3, high Ki-67 expression was significantly related to TNM stage (III/IV vs. I/II: risk ratio [RR] 2.03; 95% CI: 1.68–2.44; p < 0.001), pathological T stage (T3/T4 vs. T1/T2: RR = 1.67; 95% CI: 1.35–2.06; p < 0.001), metastasis (yes vs. no: RR = 2.15; 95% CI: 1.77–2.62; p < 0.001), and Fuhrman grade (III/IV vs. I/II: RR = 1.77; 95% CI: 1.20–2.60; p = 0.004). Some significant interstudy heterogeneity was observed in pathological T stage and Fuhrman grade, but analyses on TNM stage and metastasis did not exhibit significant heterogeneity.

### Sensitivity Analysis

A sensitivity analysis was conducted by sequential omission of individual studies. Results are shown in supplementary materials (Sensitivity analysis). The pooled HR of OS, CSS, and DFS were not significantly changed, suggesting the robustness of the results.

### Publication Bias

**Figure 3:**
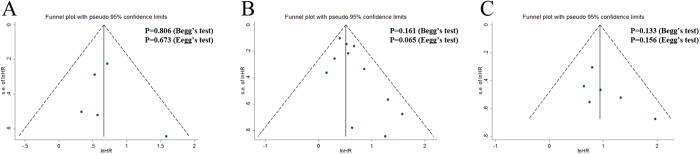
Funnel plots and Begg’s and Egger’s tests for the evaluation of potential publication bias. (**A**) Overall survival, (**B**) cancer-specific survival, and (**C**) disease-free survival.

Begg’s and Egger’s tests, as well as funnel plots, were conducted to estimate publication bias in the present meta-analysis. As shown in Fig. 3, the funnel plots indicated that the included studies had no evident asymmetry. Furthermore, the results from Begg’s test (P value) and Egger’s test (intercept with corresponding 95% CI, P value) for the included studies assessing the survival outcomes were P_Begg’s_ = 0.806, intercept 0.51 with 95% CI: −2.97 to 3.99, P_Egger’s_ = 0.673 (OS); P_Begg’s_ = 0.161, intercept 0.99 with 95% CI: −0.07 to 2.06, P_Egger’s_ = 0.065 (CSS); and P_Begg’s_ = 0.133, intercept 2.02 with 95% CI: −1.19 to 5.23, P_Egger’s_ = 0.156 (DFS). These findings suggested that significant publication bias did not exist in our meta-analysis.

## Discussion

First described in 1991 by Gerdes *et al*.^32^, Ki-67, a nuclear protein, is a famous marker of cell proliferation. It is expressed throughout the cell cycle in proliferating but not quiescent (G0) cells, so it has been used as a proliferation marker in many cancers. Recently, Ki-67 has drawn increasing attention as an attractive prognostic prediction marker and potential therapeutic target in malignant neoplasms. Several studies suggested that Ki-67 is significantly associated with the prognosis of bladder cancer, breast cancer, lung cancer, upper urinary tract urothelial carcinomas, cervical cancer, and lymphoma^6^,^9^,^33^,^34^,^35^,^36^. However, the prognostic and clinicopathological values of Ki-67 remain ambiguous in RCC. Therefore, we conducted this meta-analysis to resolve the remaining controversy and reach a reasonable conclusion.

In this study, we focused exclusively on validating Ki-67 IHC expression and evaluated the prognostic values of Ki-67 IHC expression in RCC. We concluded that high Ki-67 expression predicted unfavorable prognosis for patients with RCC. In particular, RCC patients with high Ki-67 expression exhibited poor OS, CSS, and DFS. Subgroup analysis revealed the following results. (1) In terms of ethnicity, high Ki-67 expression was correlated with poor OS, CSS, and DFS in Caucasian patients, as well as with poor DFS but not significantly with CSS in Asian patients. (2) Regarding the tumor extent, high Ki-67 expression was associated with poor OS and CSS but not significantly with DFS for all stages of RCC; with poor DFS but not significantly with OS and CSS for localized RCC; and with poor CSS for metastatic RCC. (3) For histopathological subtype, high Ki-67 expression was correlated with poor CSS and DFS for ccRCC. (4) In the cutoff of staining subgroup analysis, high Ki-67 expression was correlated with poor DFS but not significantly with CSS when the cutoff value was less than 10%. Studies with a cutoff value greater than or equal to 10% showed that high Ki-67 expression was associated with poor OS, CSS, and DFS. Subgroup analysis revealed that RCC patients with high Ki-67 expression presented a relatively unfavorable survival outcome, even though some association did not reach statistical significance. The absence of a significant association was possibly due to the relatively limited studies in the subgroups.

Our results also suggested that RCC patients with high Ki-67 expression were likely to have a higher TNM stage, pathological T stage, positive metastasis, and a higher Fuhrman grade. The biological mechanism of Ki-67 may partly explain its prognostic and clinicopathological significance in patients with RCC. Ki-67 is a well-known cell proliferation biomarker in many tumors and plays a critical role in mitosis by regulating chromatin recombinant. Ki-67 has been considered a good molecular surrogate of the aggressive behavior and therapy response for survival outcome assessment in several cancers including RCC^28^. In addition to the commonly applied proliferative activity marker, Ki-67 may be associated with promotion of epithelial-to-mesenchymal transition (EMT)^37^,^38^. EMT induction is a key process for development and progression of malignant tumors.

This study is the first comprehensive analysis on the associations between Ki-67 expression and prognostic and clinicopathological significance in patients with RCC, but several limitations should be acknowledged. First, all included studies measured Ki-67 expression via IHC, but the criteria to determine the positive or negative expression of Ki-67 were inconsistent in different studies, which may potentially contribute to heterogeneity. Therefore, a more unified standard should be defined in the future. Second, a remarkable heterogeneity of studies was observed in certain categories of analysis. The heterogeneity was probably caused by differences in factors such as the patients’ characteristics (ethnicity, nationality, gender, age, and tumor stage and grade), variation in cutoff values for Ki-67 expression, and different durations of follow up. Third, relatively few studies were extracted in some subgroup analyses, which might render premature results. With more eligible studies published in the future, an update is necessary to achieve a more reliable result. Finally, research with positive results is potentially more likely to be submitted and published than work with negative results, which could cause publication bias^39^, although this bias was not detected in the present analysis.

To conclude, despite the limitations listed above, this meta-analysis suggested the prognostic and clinicopathological importance of Ki-67 expression in RCC. The results demonstrated that high Ki-67 expression was associated with poor prognosis and advanced clinicopathological features, which could potentially serve as risk stratification markers and even therapeutic targets in RCC. However, more large-scale, multicenter prospective studies with standardized methods and long-term follow up are needed to verify our results.

## Methods

### Search Strategy

This meta-analysis was conducted in accordance with the guidelines of the Preferred Reporting Items for Systematic Reviews and Meta-Analyses (PRISMA)^40^.

A systematic literature search was performed in the electronic databases PubMed, Web of Science, and Embase on April 20, 2016 using the following search strategy: (“Ki67” or “Ki-67” or “MIB-1”) and (“carcinoma” or “neoplasm” or “tumor” or “cancer” or “malignancy”) and (“kidney” or “renal”) and (“prognosis” or “prognostic” or “survival” or “outcome” or “mortality”). Moreover, we manually searched the reference lists of relevant literature.

### Selection Criteria

Studies were included based on the following criteria: (1) the association of Ki-67 with the prognostic value in RCC should be described, (2) studies detected Ki-67 protein expression by IHC, and (3) studies reported survival outcomes (OS, CSS, or DFS) with HR and 95% CI. Exclusion criteria were as follows: (1) non-English papers; (2) case reports, letters, commentaries, meeting records, or review articles; (3) the study focused on animal models or cancer cells; (4) the study investigated the survival outcomes of RCC by the combination of Ki-67 with other IHC marker(s); (5) the study did not analyze Ki-67 protein expression, clinical features, and survival outcome; and (6) the study lacked sufficient data for obtaining HR and 95% CI. All evaluations were independently performed by three individual researchers to ensure the accurate inclusion of studies. For duplicate studies, we only retrieved the most informative and recent studies for further analyses.

### Data Extraction

The data of the eligible studies were extracted independently with a predefined form. Discrepancies in data extraction were resolved by discussion. The following data were extracted: surname of the first author, publication year, origin of the studied population, study design, extent of tumor, histopathological subtype, number of patients, gender, patient’s age, cutoff value, follow-up time, and effect estimates, namely, HR of Ki-67 expression for OS, CSS, or DFS, as well as their 95% CI (Table 1). If the HR and 95% CI were not directly available, we calculated HRs and their 95% CI based on the methods reported by Tierney *et al*.^41^.

### Quality Assessment

The quality of the included studies was evaluated using the Newcastle–Ottawa scale, which was recommended by the Cochrane Non-Randomized Studies Methods Working Group^17^. Each study can be assessed by eight methodology items with a score ranging from 0 to 9. The high scores indicated high quality. We considered studies with scores of 6 or more as high quality for the meta-analysis. Only high-quality studies were included in further analysis to assure the quality of this meta-analysis.

### Statistical Analysis

Pooled HR and RR with 95% CI were used to evaluate the association of Ki-67 expression with RCC prognosis and clinicopathological characteristics, respectively. An observed HR > 1 indicated poor prognosis for patients with high Ki-67 expression. An observed RR > 1 implied advanced clinicopathological characteristics in the high Ki-67 expression group. A heterogeneity test of pooled HR and RR was conducted using Cochran’s Q test and Higgins I-squared statistic. A P value of less than 0.1 was considered significant. The I^2^ values >50% is considered as a measure of severe heterogeneity^42^. A random-effect model was used when heterogeneity was observed (p < 0.1); otherwise, a fixed-effect model was used. The reasons for inter-study heterogeneity were also explored by using meta-regression analysis. To obtain a more precise evaluation of heterogeneity, we only performed meta-regression analysis on CSS which had sufficient included studies (n ≥ 10). Publication bias was assessed by funnel plot visual inspection and then statistically evaluated by Begg’s^43^ and Egger’s tests^44^. The statistical processes were performed by Stata 12.0 software (StatCorp, College Station, TX, USA), and p < 0.05 was considered statistically significant.

## Additional Information

**How to cite this article:** Xie, Y. *et al*. Prognostic and clinicopathological role of high Ki-67 expression in patients with renal cell carcinoma: a systematic review and meta-analysis. *Sci. Rep.* **7**, 44281; doi: 10.1038/srep44281 (2017).

**Publisher's note:** Springer Nature remains neutral with regard to jurisdictional claims in published maps and institutional affiliations.

## Supplementary Material

Supplementary Materials

## Figures and Tables

**Table 1:** Characteristics of eligible studies in the meta-analysis.

Study	Year	Country	Study design	Tumor extent^a^	Histopathological subtype	Case number	Gender (M/F)	Age (years)	Counting method	Cut-off staining	follow-up (months)	Survival analysis	Quality score*
Rioux-Leclercq	2000	France	Cohort study	all-stage	ccRCC	73	47/26	Mean 64	eyeball	20%	52	CSS	8
Yuba	2001	Japan	Cohort study	all-stage	ccRCC	52	43/9	Mean 58.4	eyeball	5.6%	39	CSS	7
Cheville	2002	USA	Cohort study	localized	ccRCC	232	NA	NA	formal counting	5%	126	CSS	8
Bui	2004	USA	Cohort study	all-stage	ccRCC	224	149/75	Mean 61.5	formal counting	10%	34	CSS	8
Kim	2004	USA	Cohort study	all-stage	ccRCC	318	215/103	Mean 61	eyeball	15%	28	CSS	8
Lehmann	2004	Germany	Cohort study	localized	ccRCC	48	27/21	Median 63	eyeball	6%	91	DFS	6
Dudderidge	2005	UK	Cohort study	localized	ccRCC + non-ccRCC	176	120/56	Mean 63.7	eyeball	12%	44	DFS	8
Kim	2005	USA	Cohort study	metastatic	ccRCC	150	107/43	Mean 59	eyeball	NA	14.8	CSS	6
Pinto	2005	Portugal	Cohort study	all-stage	ccRCC + non-ccRCC	64	34/30	Mean 61.6	eyeball	10%	86	OS	6
Kankuri	2006	Finland	Cohort study	localized	ccRCC + non-ccRCC	37	18/19	Mean 62.2	eyeball	10%	76	OS,DFS	6
Tollefson	2007	USA	Cohort study	all-stage	ccRCC	741	475/266	358/383^b^ (≥65 y/<65 y)	eyeball	≥50 positive cells/mm^2^	>39.6	CSS	9
Gontero	2008	Italy	Cohort study	all-stage	non-ccRCC	46	37/9	28/18 (≥60 y/<60 y)	eyeball	14%	84.5	OS	8
Parker	2009	USA	Cohort study	all-stage	ccRCC	634	413/221	312/322 (≥65 y/<65 y)	eyeball	≥50 positive cells/mm^2^	NA	CSS	8
Zubac	2009	Norway	Cohort study	all-stage	ccRCC	172	95/77	Mean 66.6	formal counting	10%	68.4	CSS	8
Kankuri-Tammilehto	2010	Finland	Cohort study	all-stage	ccRCC + non-ccRCC	57	NA	Mean 61	eyeball	10%	>100	OS	6
Weber	2013	Germany	Cohort study	localized	ccRCC	132	80/52	Median 63.5	eyeball	15%	122.4	CSS	8
Gayed	2014	USA	Cohort study	localized	ccRCC	401	239/162	Median 58	eyeball	10%	22	DFS,CSS	8
Teng	2014	China	Cohort study	localized	ccRCC	378	272/106	Mean 53.4	NA	50%	60	DFS	8
Zheng	2014	China	Cohort study	all-stage	ccRCC + non-ccRCC	1239	858/381	53.9	eyeball	15%	56.8	DFS	8
Virman	2016	Findland	Cohort study	all-stage	ccRCC + non-ccRCC	224	132/92	Median 65	formal counting	Median staining	64.8	OS	9

ccRCC: clear cell renal cell carcinoma; OS: overall survival; CSS: cancer-specific survival; DFS: disease-free survival; NA: not available.

^a^Reported at time of diagnosis.

^b^358 patients ≥ 65 years, and other 383 patients < 65 years.

*The quality of the included studies was evaluated using the Newcastle–Ottawa scale.

**Table 2:** Subgroup analysis of pooled HR for RCC patients with Ki-67 overexpression.

Outcome	Subgroup	Studies	Pooled HR	95% CI	P Value	Model	Heterogeneity *I*^*2*^ (%)	P Value
OS	Ethnicity							
	Caucasian	5	1.95	1.44–2.64	<0.001	fixed	0	0.594
	Asian	0	─	─	─	─	─	─
	Tumor extent							
	all-stage	4	2.01	1.46–2.77	<0.001	fixed	0	0.510
	localized	1	1.40	0.52–3.76	0.504	─	─	─
	metastatic	0	─	─	─	─	─	─
	Histopathological subtype							
	ccRCC	0	─	─	─	─	─	─
	Counting method							
	eyeball counting	4	1.86	1.22–2.82	0.004	fixed	0	0.443
	formal counting	1	2.05	1.32–3.19	0.001	fixed	─	─
	Cutoff of staining							
	<10%	0	─	─	─	─	─	─
	≥10%	4	1.86	1.22–2.82	0.004	fixed	0	0.443
	Follow up (month)							
	<60	0	─	─	─	─	─	─
	≥60	5	1.95	1.44–2.64	<0.001	fixed	0	0.594
CSS	Ethnicity							
	Caucasian	10	1.67	1.47–1.89	<0.001	fixed	12.2	0.331
	Asian	1	1.88	0.41–8.67	0.417	─	─	─
	Tumor extent							
	all-stage	7	1.80	1.52–2.13	<0.001	fixed	0	0.676
	localized	3	2.29	0.85–6.19	0.104	random	52.8	0.120
	metastatic	1	1.49	1.22–1.82	<0.001	─	─	─
	Histopathological subtype							
	ccRCC	11	1.67	1.47–1.89	<0.001	fixed	2.7	0.417
	Counting method							
	eyeball counting	8	1.66	1.44–1.90	<0.001	fixed	13.7	0.323
	formal counting	3	1.73	1.26–2.38	0.001	fixed	4.8	0.350
	Cutoff of staining							
	<10%	2	1.26	0.66–2.41	0.476	fixed	0	0.573
	≥10%	6	1.90	1.45–2.49	<0.001	fixed	19.4	0.287
	Follow up (month)							
	<60	7	1.59	1.38–1.84	<0.001	fixed	0	0.633
	≥60	3	1.93	1.22–3.03	0.005	fixed	52.5	0.122
DFS	Ethnicity							
	Caucasian	4	2.63	1.73–4.01	<0.001	fixed	15.1	0.316
	Asian	2	2.38	1.18–4.80	0.015	fixed	0	0.778
	Tumor extent							
	all-stage	1	2.11	0.74–6.50	0.178	─	─	─
	localized	5	2.63	1.79–3.85	<0.001	fixed	0	0.473
	metastatic	0	─	─	─	─	─	─
	Histopathological subtype							
	ccRCC	3	3.66	2.00–6.72	<0.001	fixed	0	0.463
	Counting method							
	eyeball counting	5	2.57	1.73–3.79	<0.001	fixed	0	0.452
	formal counting	0	─	─	─	─	─	─
	Cutoff of staining							
	<10%	1	7.18	1.91–26.99	0.004	─	─	─
	≥10%	5	2.36	1.62–3.43	<0.001	fixed	0	0.884
	Follow up (month)							
	<60	3	2.45	1.54–3.91	<0.001	fixed	0	0.653
	≥60	3	2.73	1.55–4.82	0.001	fixed	26.9	0.255

OS: overall survival; CSS: cancer-specific survival; DFS: disease-free survival; HR: hazard ratio; CI: confidence interval; RCC: renal cell carcinoma; ccRCC: clear cell renal cell carcinoma.

**Table 3:** Meta-analysis of the association between high Ki-67 expression and clinicopathological features of RCC.

Variables	Studies	Pooled RR	95% CI	P Value	Model	Heterogeneity *I*^*2*^ (%)	P Value
TNM stage (III/IV vs. I/II)	4	2.03	1.68–2.44	<0.001	fixed	18.2	0.300
pT stage (T3/T4 vs. T1/T2)	8	1.67	1.35–2.06	<0.001	random	58.5	0.018
Metastasis^a^ (yes vs. no)	5	2.15	1.77–2.62	<0.001	fixed	15.9	0.313
Fuhrman grade (III/IV vs. I/II)	8	1.77	1.20–2.60	0.004	random	95.2	<0.001

^a^Both lymph node and distant metastases; RR: relative ratio; CI: confidence interval; RCC: renal cell carcinoma.
