# Supplementary material for: Prognostic and clinicopathological role of high Ki-67 expression in patients with renal cell carcinoma: a systematic review and meta-analysis
Source: Sci Rep. 2017 Mar 13;7:44281. doi: 10.1038/srep44281 (PMC5347162; doi:10.1038/srep44281)
Supplement: Supplementary Materials [file srep44281-s1.pdf]

Prognostic and clinicopathological role of high Ki-67 expression in patients with renal cell carcinoma: a systematic review and meta-analysis

Yongpeng Xie[1,2&], Luyao Chen[1&], Xin Ma[1], Hongzhao Li[1], Liangyou Gu[1], Yu Gao[1], Yang Fan[1], Yu Zhang[1] , Xu Zhang[1]*

[&] These authors equally dedicated to this article.

[1] State Key Laboratory of Kidney Diseases, Department of Urology, Chinese PLA Medical School, Chinese PLA General Hospital, Beijing, People's Republic of China

[2] Medical School, Nankai University, Tianjin, People's Republic of China

[*] Corresponding author: Xu Zhang, M.D.

Department of Urology, Chinese PLA General Hospital, Beijing 100853, P.R. China.

Tel: +86-10-66938008; Fax: +86-10-68223575

E-mail: xzhang@tjh.tjmu.edu.cn

Supplemental table 1. Pooled HR (95% CI) of sensitivity analysis for the effect of Ki-67 expression on OS

Study Omitted	Pooled HR	95% CI	P Value	Model	Heterogeneity I^2 (%)	P Value
Pinto (2005)	1.84	1.35-2.51	<0.001	fixed	0	0.895
Kankuri (2006)	2.01	1.46-2.77	<0.001	fixed	0	0.510
Gontero (2008)	1.96	1.43-2.70	<0.001	fixed	0	0.432
Kankuri-Tammilehto (2010)	2.06	1.44-2.94	<0.001	fixed	0	0.479
Virman (2016)	1.86	1.22-2.82	0.004	fixed	0	0.443
Combined	1.95	1.44-2.64	<0.001	fixed	0	0.594

HR: hazard ratio; CI: confidence interval; OS: overall survival.

Supplemental table 2. Pooled HR (95% CI) of sensitivity analysis for the effect of Ki-67 expression on CSS

Study Omitted	Pooled HR	95% CI	P Value	Model	Heterogeneity I^2 (%)	P Value
Rioux-Leclercq (2000)	1.65	1.45-1.87	<0.001	fixed	0	0.507
Yuba (2001)	1.67	1.47-1.89	<0.001	fixed	12.2	0.331
Cheville (2002)	1.69	1.49-1.92	<0.001	fixed	2.6	0.416
Bui (2004)	1.66	1.46-1.89	<0.001	fixed	11.9	0.333
Kim (2004)	1.69	1.49-1.93	<0.001	fixed	5.7	0.389
Kim (2005)	1.80	1.53-2.11	<0.001	fixed	0	0.509
Tollefson (2007)	1.66	1.45-1.91	<0.001	fixed	12.3	0.330
Parker (2009)	1.62	1.41-1.86	<0.001	fixed	1.7	0.423
Zubac (2009)	1.65	1.45-1.87	<0.001	fixed	1.5	0.425
Weber (2013)	1.65	1.46-1.87	<0.001	fixed	0	0.559
Gayed (2014)	1.66	1.46-1.88	<0.001	fixed	5.2	0.393
Combined	1.67	1.47-1.89	<0.001	fixed	2.7	0.417

HR: hazard ratio; CI: confidence interval; CSS: cancer-specific survival.

Supplemental table 3. Pooled HR (95% CI) of sensitivity analysis for the effect of Ki-67 expression on DFS

Study Omitted	Pooled HR	95% CI	P Value	Model	Heterogeneity I^2 (%)	P Value
Lehmann (2004)	2.36	1.62-3.43	<0.001	fixed	0	0.884
Dudderidge (2005)	2.78	1.77-4.37	<0.001	fixed	0	0.505
Kankuri (2006)	2.73	1.84-4.06	<0.001	fixed	0	0.539
Gayed (2014)	2.43	1.65-3.57	<0.001	fixed	0	0.549
Teng (2014)	2.56	1.73-3.79	<0.001	fixed	0	0.452
Zheng (2014)	2.63	1.79-3.85	<0.001	fixed	0	0.473
Combined	2.56	1.79-3.68	<0.001	fixed	0	0.598

HR: hazard ratio; CI: confidence interval; DFS: disease-free survival.
